# Paediatric cranial ultrasound: abnormalities of the brain in term neonates and young infants

**DOI:** 10.1186/s13244-025-02031-4

**Published:** 2025-07-22

**Authors:** Caoilfhionn Ní Leidhin, Michael Paddock, Paul M. Parizel, Richard R. Warne, Peter Shipman, Rahul Lakshmanan

**Affiliations:** 1 Department of Medical Imaging, Perth Children’s Hospital, Perth, Western Australia Australia; 2 Perth Radiological Clinic, Perth, Western Australia Australia; 3 SKG Radiology, Subiaco, Western Australia Australia; 4 School of Medicine, The University of Notre Dame Australia, Fremantle, Western Australia Australia; 5 School of Medicine & Population Health, University of Sheffield, Sheffield, United Kingdom; 6 Medical School, University of Western Australia, Perth, Western Australia Australia; 7 Department of Radiology, Royal Perth Hospital (RPH), Perth, Western Australia Australia; 8 Perron Institute, Faculty of Medicine, Centre of Neurological and Neuromuscular Disorders, University of Western Australia, Perth, Western Australia Australia

**Keywords:** Head, Brain, Ultrasonography, Infant, Pathology

## Abstract

**Abstract:**

Cranial ultrasound is a critical screening tool in the detection of cerebral abnormalities in term neonates and infants, and is complementary to other imaging modalities. This pictorial review illustrates the diverse central nervous system pathologies which can affect the term neonatal and infantile brain, including vascular abnormalities (hypoxic ischaemic injury, perinatal arterial ischaemic stroke, cerebral sinovenous thrombosis, vein of Galen aneurysmal malformations, subpial haemorrhage, and dural sinus malformations); infections (congenital (cytomegalovirus and toxoplasmosis) and bacterial meningoencephalitis); genetic disorders and malformations (callosal agenesis, tuberous sclerosis, developmental megalencephaly, lissencephaly-pachygyria, and grey matter heterotopia); tumours (choroid plexus papilloma, atypical teratoid/rhabdoid tumour, and desmoplastic infantile glioma) and trauma (birth-related, inflicted injury). Each condition is explored with a focus on its sonographic characteristics—some have rarely, if ever, been described on ultrasound.

**Critical relevance statement:**

Through this case review, we illustrate various pathologies affecting the term neonatal and infantile brain, including vascular lesions, infection, genetic disorders/malformations, tumours and trauma: some of these pathologies have rarely, if ever, been described on CUS.

**Key Points:**

- Cranial ultrasound (CUS) is a critical screening tool for the term brain.
- Many term neonatal and infantile pathologies can be detected on CUS.
- Some of the pathologies illustrated in this paper have rarely been described on US.

**Graphical Abstract:**

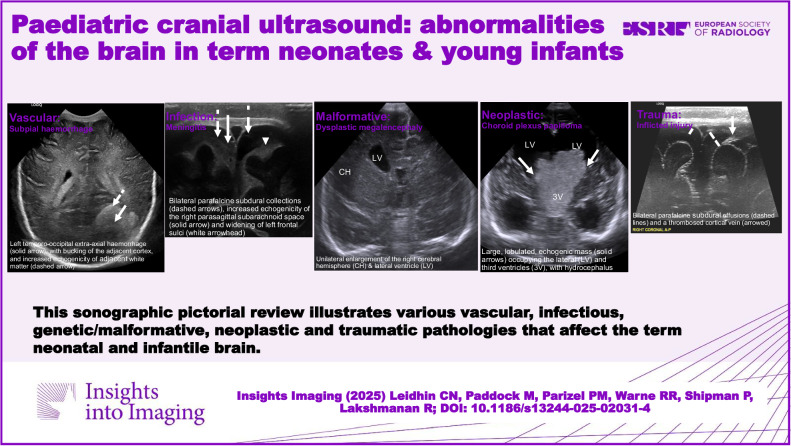

## Introduction

Cranial ultrasound (CUS) is an essential screening tool for the neonatal and infantile brain. Through this comprehensive sonographic pictorial review, we illustrate various vascular, infectious, genetic/malformative, neoplastic and traumatic processes which affect the term neonatal and infantile brain.

## Vascular abnormalities

### Hypoxic ischaemic encephalopathy

Hypoxic ischaemic encephalopathy (HIE) or hypoxic ischaemic injury (HII) following perinatal asphyxia is a major cause of morbidity and mortality [1]. Two main forms of hypoxia occur: severe, acute, profound hypoxia (e.g., due to abruptio placenta), which preferentially affects the highly metabolic, actively myelinating basal ganglia and thalami; and prolonged, partial hypoxia (e.g., in prolonged, difficult labour), which results in a peripheral/parasagittal/borderzone/“watershed” pattern of injury, preferentially affecting the cortex and subcortical white matter of the parieto-occipital and posterior temporal lobes [2, 3]. HIE is initially graded clinically as mild, moderate or severe: the clinical grade is combined with the electroencephalogram and neuroimaging findings to estimate disease severity and predict neurodevelopmental outcome [4].

**Fig. 1:**
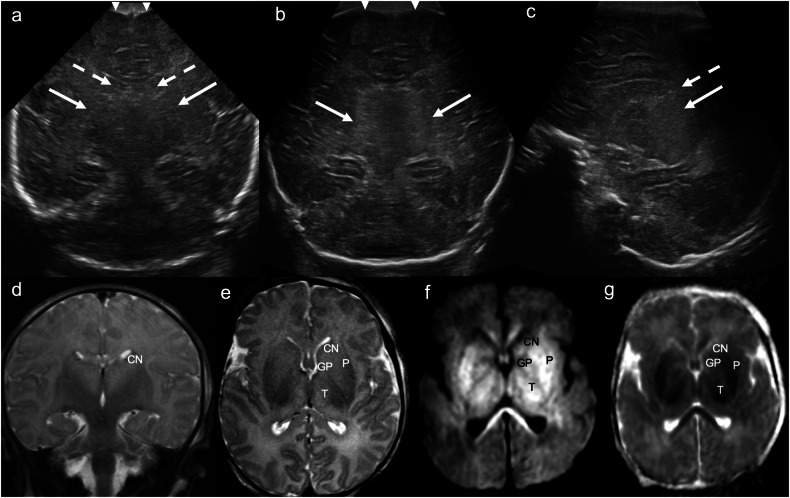
Hypoxic ischaemic injury. Day 1 ultrasound in a term neonate with cord prolapse, poor APGAR scores, and seizures at birth. **a**, **b** Coronal images demonstrate “slit-like” lateral ventricular frontal horns (dashed arrows), diffuse effacement of extra-axial CSF spaces (white arrowheads), and bilateral, symmetrical increased echogenicity of the basal ganglia (**a**) and thalami (**b**) (solid arrows). **c** Parasagittal image demonstrates an effaced lateral ventricle (dashed arrow) and increased echogenicity and swelling of the thalamus (solid arrow). **d** Coronal and (**e**) axial T2WI, (**f**) axial trace DWI and (**g**) ADC map demonstrate corresponding bilateral, symmetrical T2 hyperintensity and swelling (**d**, **e**) and restricted diffusion (high signal on DWI, low signal on corresponding ADC map) (**f**, **g**) involving the caudate nuclei, lentiform nuclei (globi pallidi and putamina), and thalami

While magnetic resonance imaging (MRI) has emerged as the gold-standard imaging technique for suspected HIE, CUS is a valuable screening tool in infants admitted to the neonatal intensive care unit with encephalopathy [5], especially given its ability to monitor changes over time. Early (i.e., within 12 h of insult) CUS findings suggestive of HIE include: cerebral oedema with ventricular and sulcal effacement, and reduced grey-white differentiation [1, 6]. There may also be increased echogenicity of the subcortical and periventricular white matter [6]. Bilateral, symmetrical increased echogenicity of the deep grey nuclei develops gradually 2–4 days after the acute insult (Fig. 1) [1, 6]. The “four-column sign” refers to increased echogenicity of bilateral putamina and thalami and indicates moderate to severe HIE [1, 6]. Cortical necrosis, seen on US as thickening and increased echogenicity of the cortex, may be seen toward the end of the first week [6]. Resistive indices (RI) on Doppler assessment can be abnormal and are usually low. A RI ≤ 0.62—obtained with Doppler US during the first 24 h of life in the anterior, middle cerebral and basilar arteries—has been shown to differentiate normal from clinically asphyxiated neonates (any grade) with up to 95–99% accuracy [7]. Outcomes in HIE are poor, with up to 50% mortality and permanent neurological deficits in 60% of survivors [8].

### Perinatal arterial ischaemic stroke

Perinatal arterial ischaemic stroke, defined as stroke occurring between 20 weeks’ gestation and 28 days of life, is the most common cause of paediatric stroke and a leading cause of cerebral palsy [9]. This typically manifests in infants as seizures [10]. Risk factors can be categorised into: perinatal disorders, i.e., congenital heart disease, coagulopathies, vasculopathies, infection, and dehydration; maternal disorders, i.e., pre-eclampsia, autoimmune disease, and coagulopathies; and placental disorders, i.e., thrombosis, abruption, and infection [11]. The middle cerebral artery territory is most commonly affected, more often the left, which likely reflects placental and/or systemic venous emboli preferentially entering the left common carotid artery (and thus the left anterior cerebral circulation) via a right-to-left shunt (patent foramen ovale, patent ductus arteriosus, atrial or ventricular septal defects) [11].

**Fig. 2:**
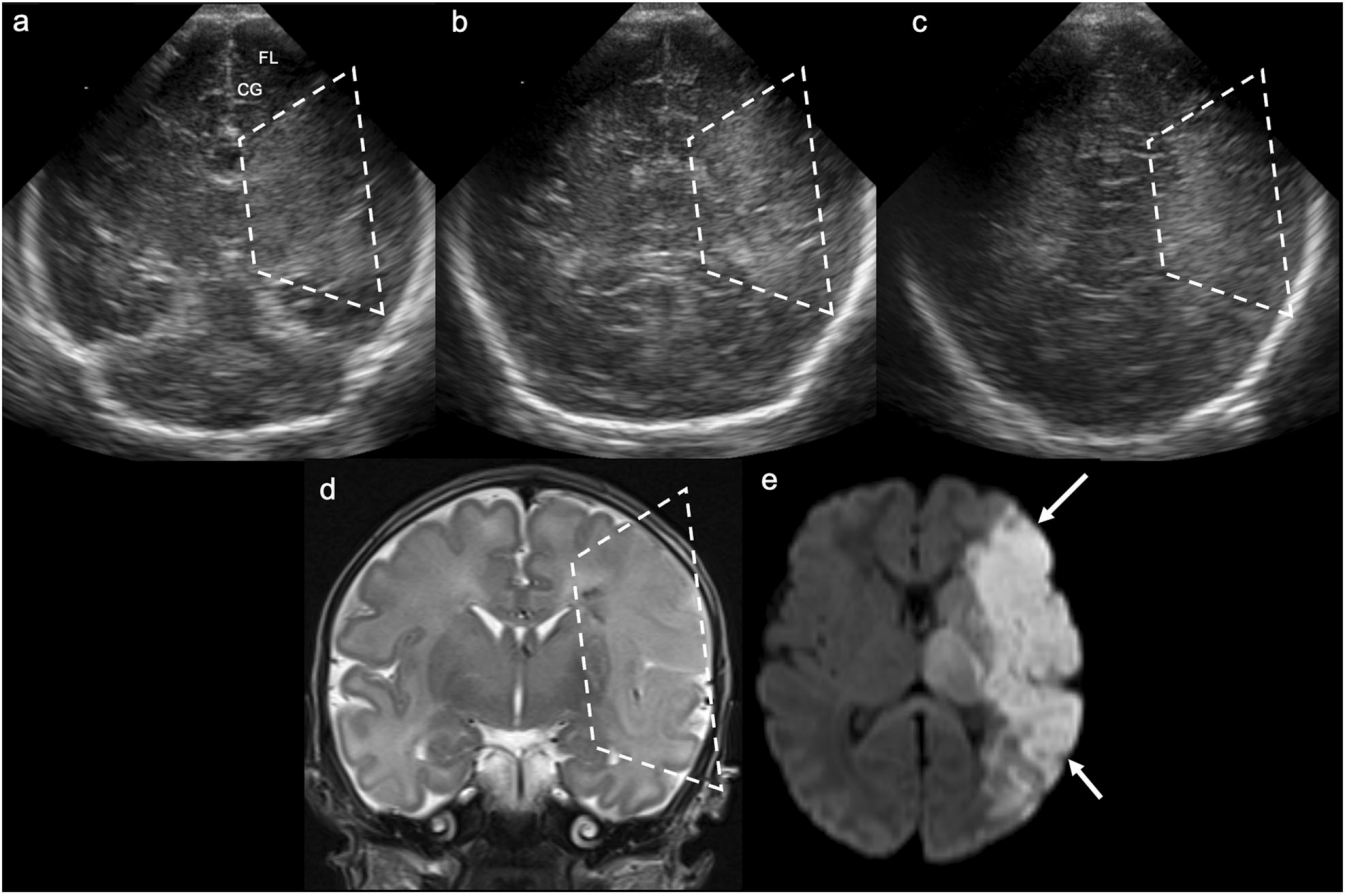
Perinatal arterial ischaemic stroke. Day 2 ultrasound in a 36 + 1-week gestation neonate with seizures. **a**–**c** Coronal images at the level of the third ventricle (**a**), lateral ventricular trigones (**b**), and occipital lobes (**c**) demonstrate ill-defined, wedge-shaped increased echogenicity in the left cerebral hemisphere (dashed lines), sparing the medial left frontal lobe (FL) and cingulate gyrus (CG); there is associated brain swelling with loss of sulcation. **d** Coronal T2WI demonstrates corresponding hyperintensity in the left cerebral hemisphere, involving both cortex and subcortical white matter, with loss of normal grey-white matter differentiation (dashed lines). **e** Axial trace DWI demonstrates restricted diffusion (ADC map not shown) in the left cerebral hemisphere corresponding to the left middle cerebral artery territory (solid arrows)

CUS may be the first modality to suggest arterial ischaemic stroke (AIS). Initial “normal” appearances within the first few days of life can be misleading [12]: serial/repeat scanning is advocated given that sensitivity increases over time, from up to 83% (by an expert evaluator) within the first 24 h to up to 93% after 48 h [13]. On day 1, AIS appears as subtle, ill-defined increased echogenicity in the affected arterial territory [6]. The characteristic US finding of a triangular/wedge-shaped area of increased echogenicity, which extends to the brain surface, with loss of grey-white matter differentiation, does not develop until 24–72 h post-insult (Fig. 2) [6, 14]. Doppler abnormalities seen on CUS in AIS include: lack of blood flow in the affected vessel; luxury perfusion with asymmetrically increased systolic velocities in the affected territory; asymmetrically increased diastolic velocities and thus decreased resistive indices; and an increased number and size of vessels at the periphery of the infarct on power Doppler [15]. MRI with diffusion-weighted imaging (DWI) is essential to confirm the diagnosis. Over 2–4 weeks, CUS demonstrates tissue loss with cystic encephalomalacia and ex vacuo ventricular dilatation [6, 16].

### Neonatal cerebral sinovenous thrombosis

Neonatal cerebral sinovenous thrombosis (CSVT) is a further important cause of perinatal stroke, associated with poor neurodevelopmental outcome in 50% of affected neonates [17]. CSVT commonly manifests with seizures and/or apnoeas. Risk factors include: infection/sepsis, dehydration, hypercoagulability and perinatal insult/trauma [16]. In approximately 50% of cases, multiple sinuses are involved. When isolated, the superior sagittal, straight, and transverse sinuses are the most commonly affected in descending order of frequency [18].

**Fig. 3:**
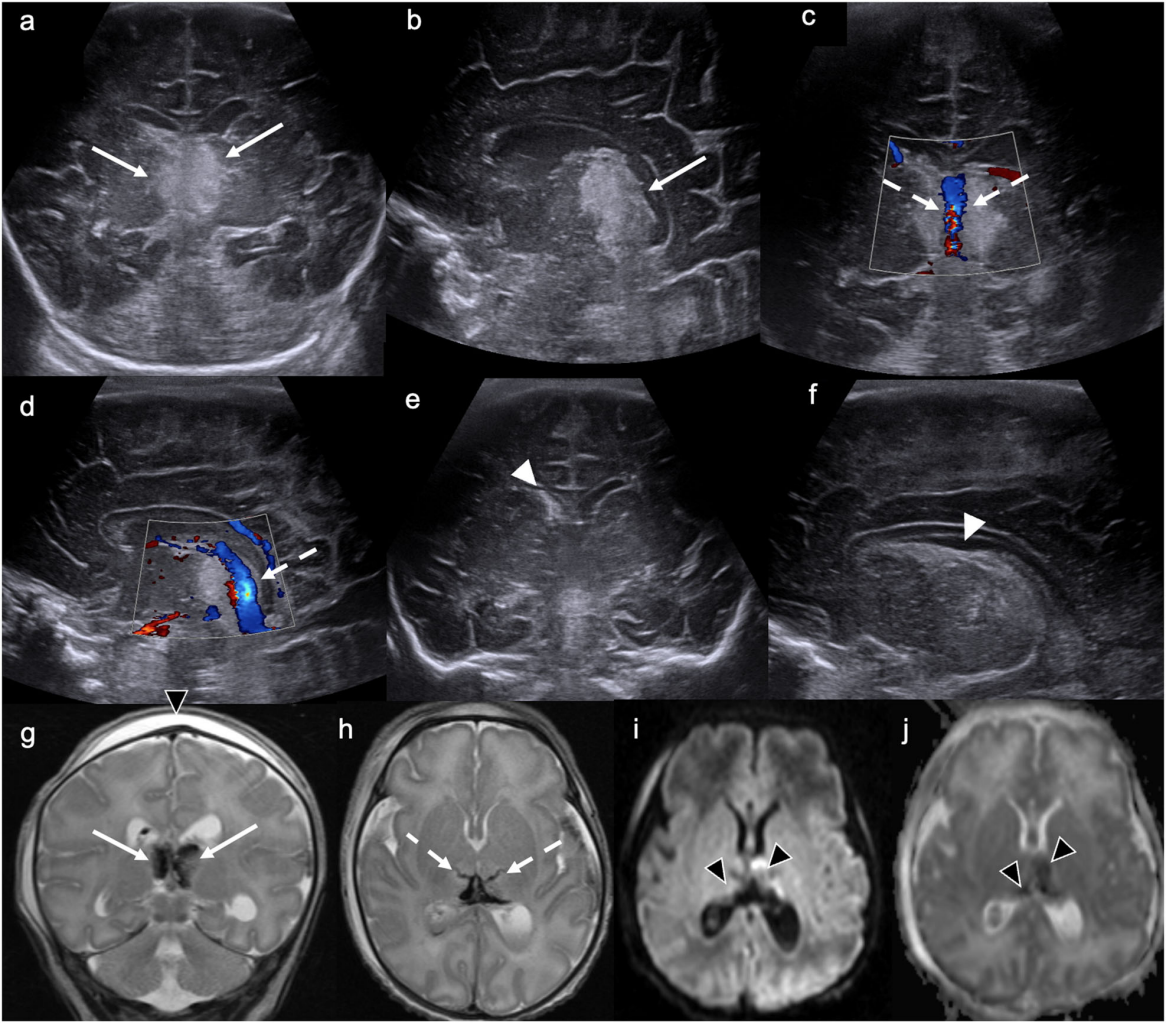
Cerebral sinovenous thrombosis. Day 2 ultrasound in a 36-week gestation neonate with difficulty feeding after a complicated (failed forceps) delivery. **a** Coronal and (**b**) left parasagittal images demonstrate bilateral, slightly asymmetrical (left > right) increased echogenicity of the medial thalami (solid arrows). **c** Coronal and (**d**) left parasagittal colour Doppler images demonstrate flow in the internal cerebral veins (dashed arrows), suggesting patency. **e** Coronal image demonstrates echogenic material in the right frontal horn, partially effacing it (white arrowhead). **f** Right parasagittal image confirms echogenic material in the caudothalamic groove (white arrowhead), consistent with germinal matrix haemorrhage. This constellation of findings—bilateral thalamic increased echogenicity, presumed haemorrhagic infarction, and right grade 1 intraventricular haemorrhage—is suggestive of cerebral sinovenous thrombosis. **g** Coronal T2WI demonstrates corresponding bilateral hypointensity in the medial thalami, confirming haemorrhage (solid arrows). Note the subgaleal collection secondary to traumatic delivery (black arrowhead). **h** Axial T2WI demonstrates linear low signal in bilateral perforator veins of the thalamus, consistent with thrombosis (dashed arrows), which was not evident on CUS. **i** Axial trace DWI and (**j**) ADC map demonstrate restricted diffusion in bilateral thalami (left > right) (black arrowheads), consistent with acute thalamic haemorrhagic venous infarction

US findings suggestive of CSVT include: intraventricular haemorrhage; parenchymal haemorrhagic infarction involving the thalamus (often unilateral), basal ganglia, parasagittal cortex, and bilateral frontal lobes; as well as periventricular congestion [17, 18]. The lumen of the affected sinus(es) may be distended with hyperechoic thrombus [15], and the Doppler flow signal within the affected sinus(es) may be absent or decreased (Fig. 3) [17]. Although ever-improving Doppler techniques facilitate more detailed assessment of the cerebral venous system [19], MRI or computed tomography (CT) venography should be performed if the CUS appears normal but there remains ongoing clinical suspicion of CSVT.

### Vein of Galen aneurysmal malformation

Vein of Galen aneurysmal malformations (VoGM), also known as median prosencephalic arteriovenous fistulas, are rare congenital vascular anomalies. Lasjaunias described two types of VoGMs: choroidal and mural [20]. However, they have more recently been categorised by Hauck et al [21] into: mural simple (type I), mural complex (type II), choroidal (type III), and choroidal with deep venous drainage (type IV). Choroidal VoGMs consist of multiple arteriovenous fistulas between choroidal and/or transmesencephalic collicular feeding arteries and a dilated median prosencephalic vein (of Markowski), the embryological precursor of the vein of Galen. Choroidal VoGMs are more common and clinically more severe than mural VoGMs. Affected neonates present early with high-output cardiac failure due to significant arteriovenous shunting. Mural VoGMs comprise a “single hole” fistula within the wall of the median prosencephalic vein. They tend to have less arteriovenous shunting and pose a lower risk of heart failure. Patients typically present later in infancy/childhood with hydrocephalus [21–23], which results from a combination of venous hypertension and disturbed CSF absorption, intraventricular haemorrhage and mechanical obstruction, i.e., aqueductal stenosis [24].

**Fig. 4:**
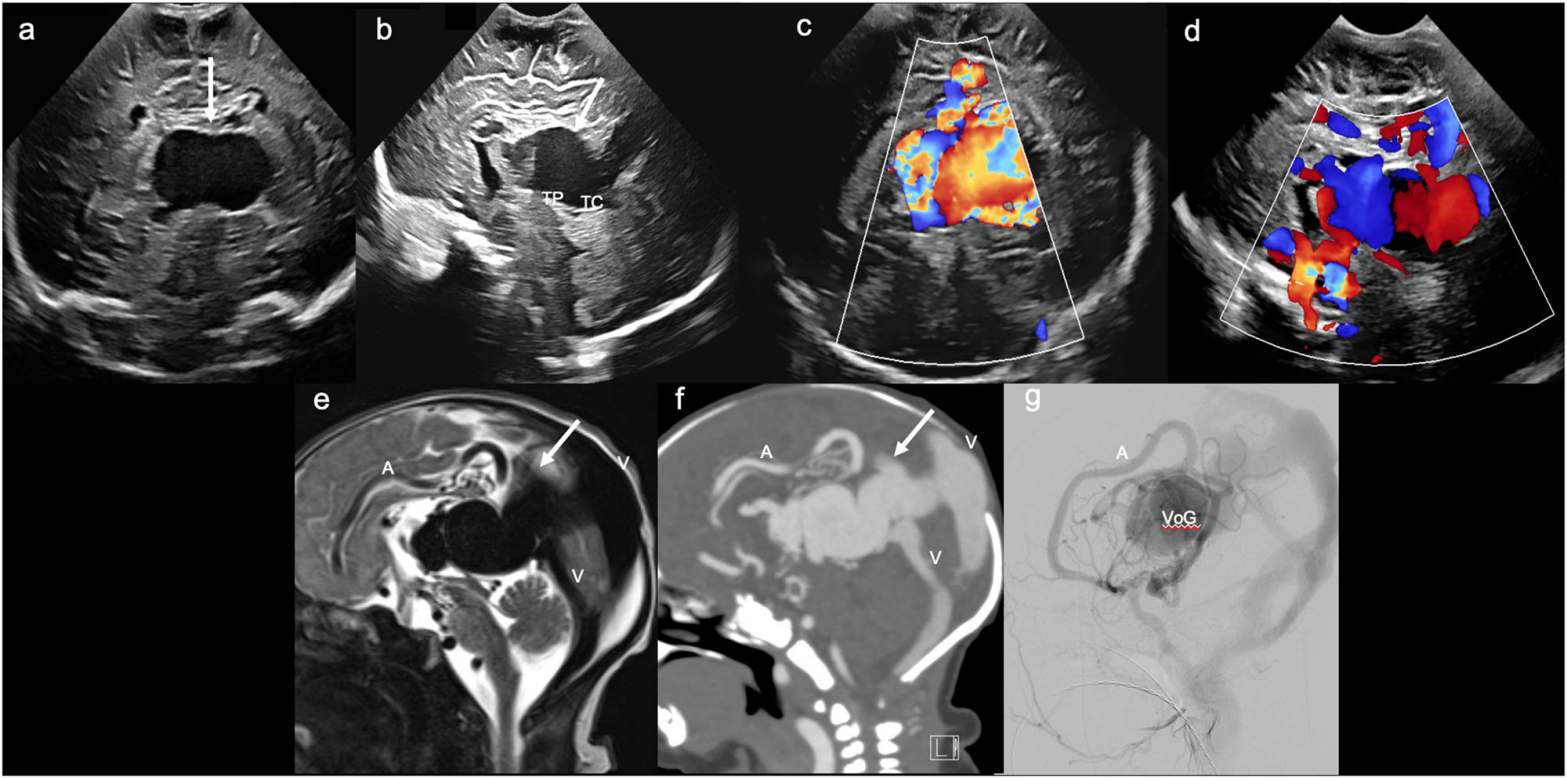
Vein of Galen aneurysmal malformation. Day 1 ultrasound in a term neonate with high-output cardiac failure. **a** Coronal image demonstrates a central, ovoid, anechoic mass (solid arrow). **b** Midline sagittal image confirms that the anechoic mass (solid arrow) is centred on the pineal recess, lying posterior to the tectal plate (TP) and superior to the tentorium cerebelli (TC). **c** Coronal and (**d**) midline sagittal colour Doppler images demonstrate marked vascularity within the sac, with aliasing artefact suggesting turbulent flow. **e** Midline sagittal T2WI and (**f**) contrast-enhanced CT demonstrate a vascular mass, with T2 low signal flow voids (**e**) and avid contrast enhancement (**f**). Large arterial feeders (A) and draining venous channels (V) are evident. **g** Midline sagittal digital subtraction angiography (DSA) demonstrates arteriovenous shunting of blood from a large anterior cerebral artery (A) to an enlarged/aneurysmal median prosencephalic vein (VoG)

VoGMs are typically diagnosed on antenatal or postnatal US [25]. The characteristic sonographic findings include a large, round/ovoid, anechoic, midline lesion, located posterior to the third ventricle/tectal plate and superior to the cerebellum, with turbulent blood flow on Doppler (Fig. 4) [26, 27]. Doppler can also demonstrate the enlarged arterial feeders (with elevated velocities and reduced RIs compared with non-involved arteries), as well as the enlarged draining sinus (with elevated velocities) [15]. VoGMs typically result in ventricular dilatation, which is easily assessed on US, as well as parenchymal injury, with volume loss particularly involving the white matter [28].

The gold-standard treatment for most VoGMs is endovascular embolisation. MRI brain and venography are crucial in clarifying the venous drainage (especially that of the internal cerebral veins) prior to embolisation to avoid major neurological complications [29]. Doppler US may be of value in patient management and follow-up, as research has demonstrated that (abnormally low) RIs normalise following successful embolisation [30], reflecting restoration of normal vascular resistance. This also facilitates monitoring for recurrence or residual shunting, given that decreasing RI could represent recanalisation or formation of new collateral vessels. Outcomes vary significantly depending on lesion type. Mural simple lesions have a benign course and may even resolve spontaneously. Mural complex and choroidal lesions often require emergency intervention due to complications associated with high-flow arteriovenous shunting: heart failure, pulmonary hypertension, etc. There is no established treatment for type IV lesions, where progressive loss of brain substance results in a “melting brain” and ultimately death [21].

### Subpial haemorrhage

Subpial haemorrhage is an entity that, until recently, was not widely recognised. It refers to an extra-axial haemorrhage between the innermost meningeal layer, the pia mater, and the outermost cerebral cortex, the glia limitans. It is thought that injury to the glia causes stretching/rupture of perforating cortical vessels, leading to subpial haemorrhage, which obstructs cortical venous outflow, resulting in cortical and subcortical venous infarction [31]. It is typically seen in neonates and infants. It occurs in the supratentorial compartment, most often in the temporal lobes, and may be multifocal [32, 33].

**Fig. 5:**
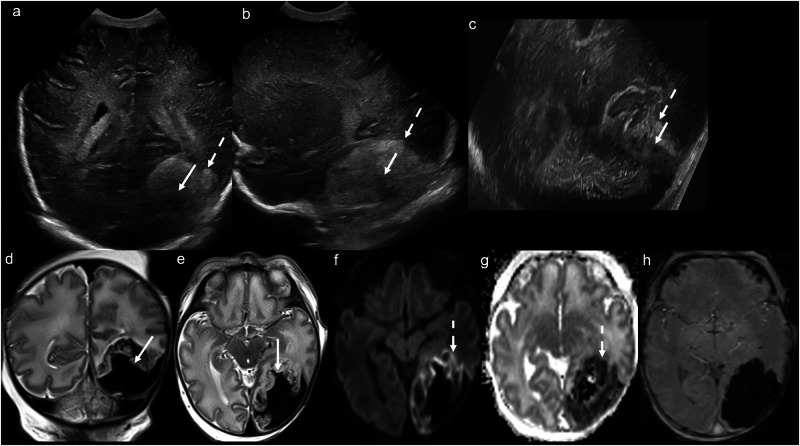
Subpial haemorrhage and haemorrhagic venous infarction. Day 1 ultrasound in a 35-week gestation neonate with non-immune hydrops fetalis, screening for congenital infection or ischaemia. **a** Coronal and (**b**) parasagittal transfontanellar, and **c** transmastoid images demonstrate an ovoid/ellipsoid hypoechoic extra-axial collection in the left posterior temporal/occipital region, which buckles the adjacent cortex (solid arrows). There is increased echogenicity of the adjacent white matter (dashed arrows). The sonographic “yin yang” sign is illustrated here. **d** Coronal and (**e**) axial T2WI confirm the large left temporo-occipital subpial haemorrhage buckling the adjacent cortex (solid arrows). **f** Axial trace DWI and (**g**) ADC map demonstrate diffusion restriction in the surrounding haemorrhagic venous infarction (dashed arrows). **h** Axial SWI demonstrates pronounced susceptibility artefact related to the haemorrhage

In the literature, the terms subpial and subarachnoid haemorrhage have been used interchangeably, as the discrete meningeal layers cannot be differentiated on US [6]. However, subpial haemorrhage has characteristic imaging findings with features of both subdural (SDH) and subarachnoid (SAH) haemorrhage. On CUS, it is visualised as an ovoid/ellipsoid superficial haemorrhage. Its outer margin conforms to the inner table of the skull (similar to SDH), yet its inner margin conforms to the sulci (similar to SAH), buckling the cortex. It is commonly associated with increased echogenicity of the underlying white matter due to venous infarction (Fig. 5) [32]. MRI demonstrates the same features as CUS but better delineates the extent of the haemorrhage, associated venous infarction and medullary venous congestion [31, 33, 34]. The “yin yang” sign on T2WI describes the low signal subpial haemorrhage juxtaposed against the high signal (compressed and infarcted) cerebral cortex/subcortical white matter [34]. There is often concomitant subarachnoid and/or parenchymal haemorrhage [33]. While subpial haemorrhage is typically associated with only minor neurological deficits, extremely or very preterm babies may have poor clinical outcomes [35].

### Dural sinus malformation

Dural sinus malformations (DSM) are rare congenital vascular anomalies, whereby antenatal arteriovenous shunting results in massive dilation of one or more dural venous sinuses [36]. Lasjunas classified these as either posterior (involving the posterior sinuses and/or torcular Herophili/venous sinus confluence) or lateral (involving the sigmoid sinus or jugular bulb) [37]. In torcular DSMs, a giant “lake” replaces the torcular Herophili/venous sinus confluence. These can be graded from I to IV depending on the degree of arteriovenous shunting. Grade I DSMs have no arterial feeders and carry the best prognosis. Grade IV postnatal DSMs contain multiple arterial feeders and are associated with brain damage and mortality of up to 75% [38]. DSMs can be misdiagnosed as VoGMs.

Torcular DSMs are usually detected on antenatal or postnatal CUS as large, hypoechoic or anechoic masses in the torcular region [38], i.e., posterior to the cerebellum (c.f. VoGMs, which are superior). Colour Doppler demonstrates vascularity at the margins of the lesion with minimal, slow internal flow [39] (Supplementary Fig. 1). Grade IV lesions are associated with parenchymal brain injury, e.g., leukomalacia and/or haemorrhagic infarction, and there may be associated hydrocephalus and crowding of the posterior fossa. Low-grade lesions often spontaneously resolve. Intermediate-grade lesions are treated with endovascular embolisation. A conservative approach is recommended in postnatally diagnosed high-grade cases due to anticipated poor outcomes [38].

## Infections

### Congenital cytomegalovirus infection

Cytomegalovirus (CMV) infection is one of the most common congenital infections and results from transplacental transmission [40]. It is a notable cause of morbidity in children with long-term sequelae (e.g., sensorineural hearing loss and/or neurological sequelae) in up to 32% of infants following maternal infection during the first trimester [41]. All neonates with CMV infection require neuroimaging, and CUS is commonly performed in the first instance. Imaging findings depend on the timing of infection:

Infection early in gestation: Cortical malformations, e.g., lissencephaly and polymicrogyria, cerebral atrophy, cerebellar dysgenesis, white matter injury (manifesting as periventricular or parenchymal calcification) and/or ventriculomegaly.

Later infection: White matter and ventricular inflammation, with periventricular/subependymal cysts, and ventricular septa [42].

**Fig. 6:**
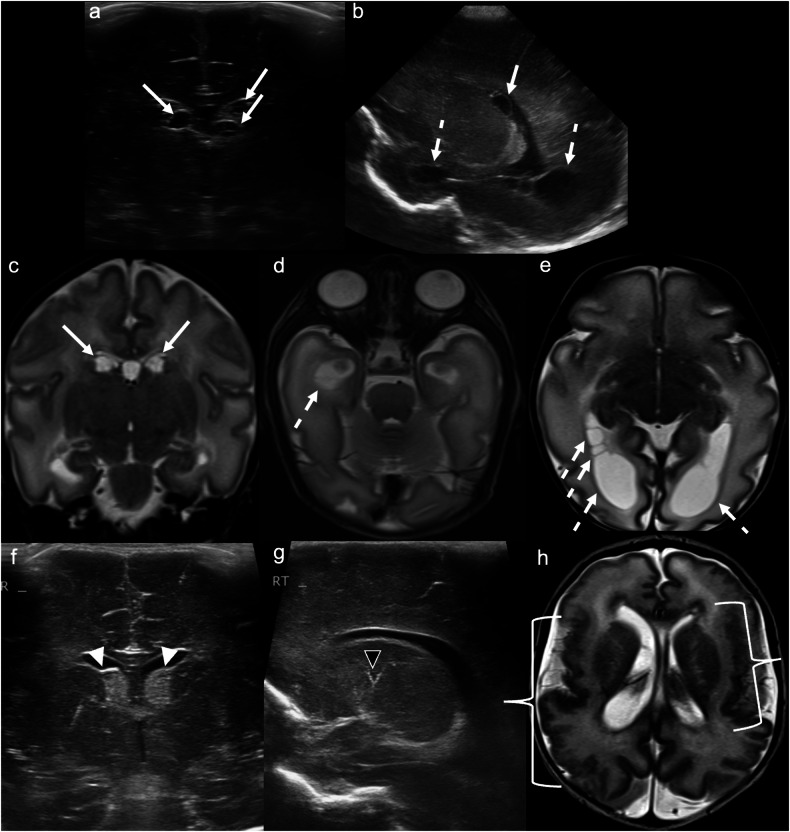
Congenital cytomegalovirus infection. Day 1 ultrasound in a 31 + 6-week gestation neonate, to assess for intraventricular haemorrhage. **a** Coronal image demonstrates multiple, bilateral septated subependymal/caudothalamic groove cysts (solid arrows) of mixed echogenicity. **b** Right parasagittal image shows a subependymal cyst extending posterior to the foramen of Monro (solid arrow), as well as periventricular cysts adjacent to the temporal and occipital horns (dashed arrows). **c** Coronal and (**d**, **e**) axial T2WI confirm multiple subependymal/caudothalamic groove (solid arrows) and periventricular (dashed arrows) cysts. Companion case: Day 1 ultrasound in a different 32-week gestation neonate. **f** Coronal image demonstrates bilateral echogenic subependymal/caudothalamic groove cysts (white arrowheads). **g** Right parasagittal image reveals linear, branching echogenicity within the basal ganglia/thalami, consistent with lenticulostriate mineralisation (black arrowhead). **h** Axial T2WI demonstrates bilateral polymicrogyria (brackets)

Thus, common CUS findings include: periventricular and cortical/subcortical calcifications; lenticulostriate vasculopathy; increased white matter echogenicity; germinolytic cysts; and ventriculomegaly [43]. Periventricular cysts in the anterior temporal lobe are particularly suggestive of CMV (Fig. 6) [44]. While CUS and MRI are complementary in the diagnostic assessment of congenital CMV infection, MRI is superior in the detection of white matter abnormalities [45].

### Congenital toxoplasmosis infection

**Fig. 7:**
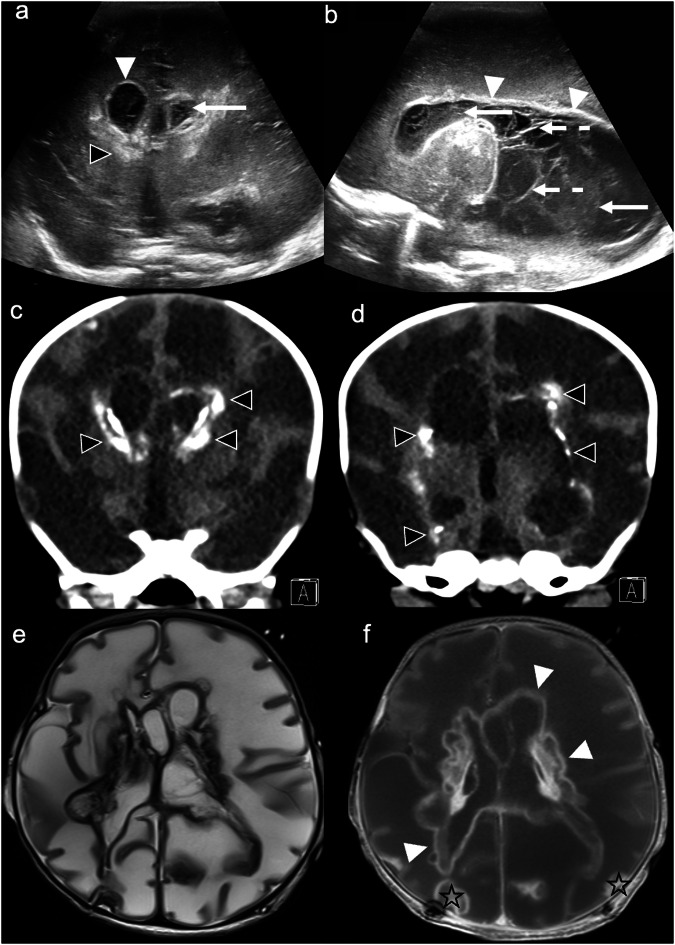
Congenital toxoplasmosis infection. Day 13 ultrasound in a term neonate with seizures and a bulging anterior fontanelle. **a** Coronal and **b** parasagittal images demonstrate severe ventriculitis with intraventricular debris (solid arrows), septations (dashed arrows), ventriculomegaly and ependymal thickening/increased echogenicity (white arrowheads); there is also periventricular echogenicity with posterior acoustic shadowing indicating calcification (black arrowhead), as well as diffuse brain swelling and diffusely increased parenchymal echogenicity with reduced grey-white differentiation. **c**, **d** Coronal CT images demonstrate coarse bilateral periventricular calcifications (black arrowheads). **e** Axial T2WI demonstrates diffuse parenchymal high signal and brain swelling, consistent with cerebritis. **f** Axial post-contrast T1WI demonstrates diffusely thickened, enhancing ependyma indicating ventriculitis (white arrowheads), along with bilateral parietal rim-enhancing lesions, consistent with abscesses (*)

Toxoplasmosis, caused by the protozoan parasite *Toxoplasma gondii*, is typically acquired through ingestion or handling of cysts in undercooked meat or oocytes excreted by cats. Pregnant women who develop toxoplasmosis can transmit the infection transplacentally [46]. Similar to CMV, infections early in pregnancy are more severe [47]:

Infection before 20 weeks’ gestation: Hydrocephalus, cerebral and cerebellar volume loss and parenchymal destruction, and diffuse large calcifications (Fig. 7) [48].

After 20 weeks: Smaller (periventricular and parenchymal) calcifications, less frequent hydrocephalus.

Hydrocephalus can be: obstructive (at the level of the cerebral aqueduct and/or foramina of Monro) due to either ventricular inflammation (ventricular septations/debris) or periventricular calcification; or non-obstructive, reflecting abnormal CSF absorption/fibrosis in the context of ventricular and leptomeningeal inflammation [49]. Parenchymal calcifications likely reflect focal areas of brain necrosis [48]. Unlike CMV, cortical malformations are uncommon in congenital toxoplasmosis [48].

### Bacterial meningitis

**Fig. 8:**
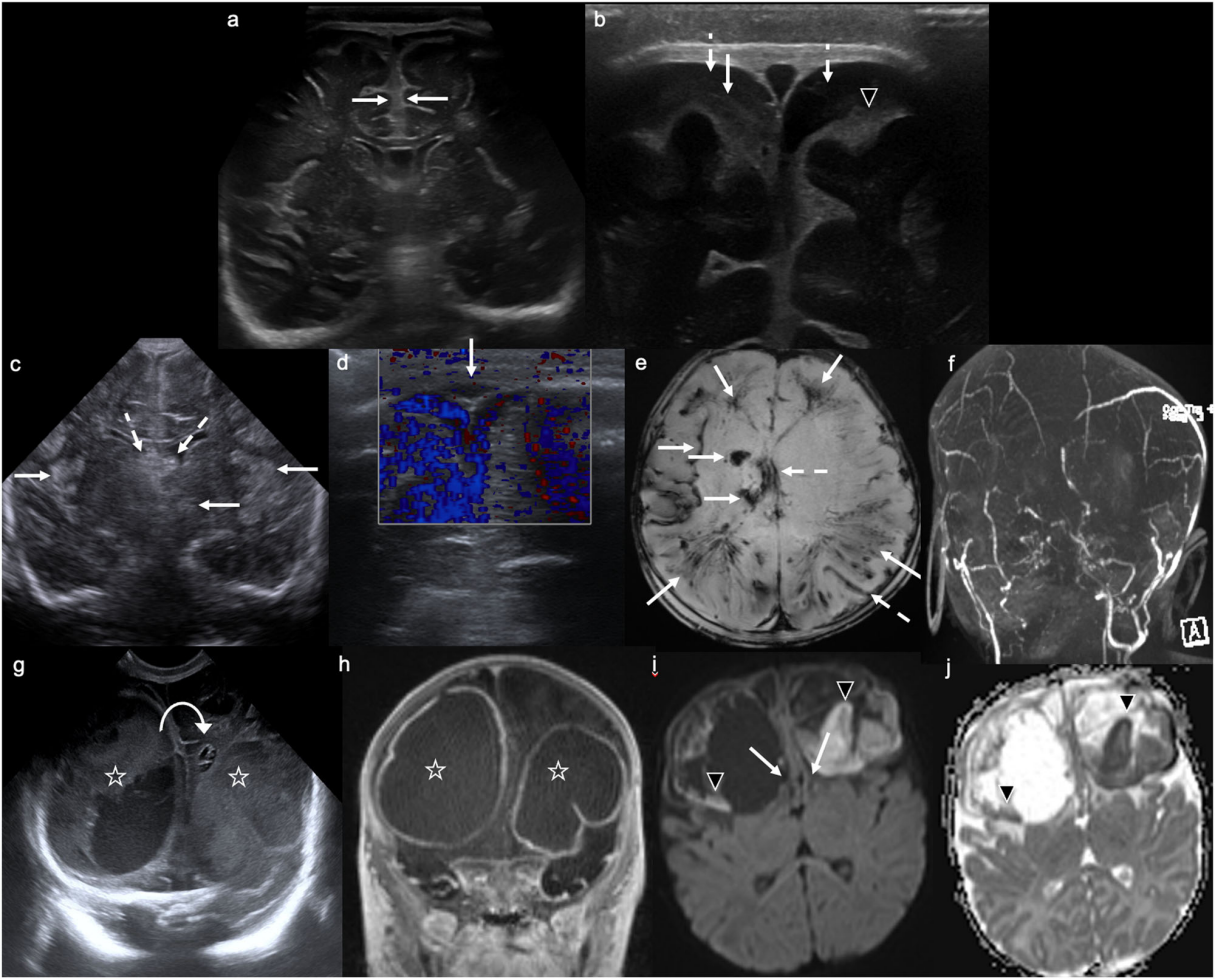
Bacterial meningitis. Ultrasound at 4 months in a drowsy infant with a bulging anterior fontanelle. **a** Coronal image demonstrates increased echogenicity in the interhemispheric subarachnoid space (solid arrows). **b** Coronal image with a high-frequency linear probe reveals bilateral hypo-/anechoic parafalcine subdural collections (left > right) (dashed arrows), increased echogenicity of the right parasagittal subarachnoid space (solid arrow), and widening of left frontal sulci (black arrowhead) due to distension of the subarachnoid space by purulent material. Findings are consistent with bacterial meningitis—caused by *Haemophilus influenzae* in this case. Ultrasound at 3 weeks in a neonate presenting with septic shock. **c** Coronal image demonstrates ill-defined, bilateral (left > right), multifocal cortical, subcortical, and deep grey matter increased echogenicity (solid arrows). There is also echogenic material in the frontal horns of both lateral ventricles, suggesting purulent/infectious debris or intraventricular haemorrhage (dashed arrows). **d** Coronal colour Doppler image demonstrates absence of flow in the superior sagittal sinus (solid arrow). **e** Axial SWI reveals corresponding bilateral susceptibility artefact, consistent with haemorrhage, involving the right insular cortex, bilateral frontal and parietal subcortical white matter, and the right corpus striatum (solid arrows); linear susceptibility artefact represents cortical and medullary vein thrombosis (dashed arrows). **f** Coronal MRV demonstrates a lack of signal in most of the dural venous sinuses (superior sagittal, transverse, and sigmoid), consistent with extensive dural venous sinus thrombosis. Findings are consistent with meningoencephalitis—due to Group B *Streptococcus* sepsis in this case, with extensive secondary haemorrhagic venous infarction. Ultrasound at 28 days in a term neonate presenting with desaturations and cyanosis. **g** Coronal image demonstrates large, bilateral ovoid/“box”-shaped frontal lobe masses, right > left (*), with mixed internal echogenicity, consistent with abscesses. Minor leftward midline shift is present (curved arrow). **h** Coronal post-contrast T1WI details thick rim enhancement of bilateral frontal lobe abscesses (*). **i** Axial trace DWI and **j** ADC map show globular restricted diffusion within the abscesses, indicating pus (black arrowheads); note the effacement of the frontal horns of both lateral ventricles due to mass effect (solid arrows). Findings are consistent with bilateral frontal lobe abscesses, due to *Citrobacter* infection in this case

Bacterial meningitis typically arises from bacteraemia, seeding of the choroid plexus and CSF, and ventricular and meningeal inflammation [50]. Group B *Streptococcus* and *E. coli* are common causative organisms of neonatal bacterial meningitis [51]. While bacterial meningitis is a clinical and microbiological diagnosis [52], CUS can support its diagnosis and monitor for complications thereof. US findings include: widening and increased echogenicity of cortical sulci (indicating meningeal inflammation, the earliest and most common finding); extra-axial collections, often echogenic and/or septated (indicating empyema); intraventricular debris/septations and thickened, echogenic, irregular ependyma (indicating ventriculitis); ventricular dilatation; and abnormal, increased parenchymal echogenicity (indicating cerebritis and/or abscess formation; see below) [6, 50, 53]. Imaging with a high-frequency linear array transducer is crucial for detecting small subdural effusions [53] (Fig. 8a, b).

Group B *Streptococcus* is associated with vascular complications [54], necessitating screening for arterial and haemorrhagic venous infarctions in the setting of confirmed infection (Fig. 8c–f). One of the most devastating complications of bacterial meningitis is brain inoculation and infection. Early, non-encapsulated parenchymal infection, e.g., cerebritis, can evolve to focal, suppurative abscess formation. *Citrobacter koseri*, although a rare cause of neonatal meningitis, results in cerebral abscess formation in up to 76% of cases [55]. Abscesses are typically visualised on CUS as well-defined, complex lesions with hyperechoic, hypervascular margins, central hypoechogenicity [50, 53] and sometimes, a hypoechoic halo [56]. *Citrobacter* abscesses have a characteristic “polygonal” shape (Fig. 8g–j) [57]. Despite advances in preventive and critical care medicine, neonatal bacterial meningitis can result in significant mortality and neurological deficits [58].

## Genetic disorders and malformations

### Callosal agenesis

Callosal agenesis or dysgenesis describes abnormal commissuration leading to either a complete (agenesis) or partial (dysgenesis) developmental anomaly of the corpus callosum. It may be associated with various neurological symptoms and signs, from mild to devastating, and is often accompanied by other brain malformations.

Agenesis or dysgenesis of the corpus callosum can be a challenging US diagnosis to make antenatally [59]. It is best detected on midline sagittal and coronal imaging [60–62], with direct and/or indirect findings:

Direct:

- absence or abnormal truncation of the corpus callosum.

**Fig. 9:**
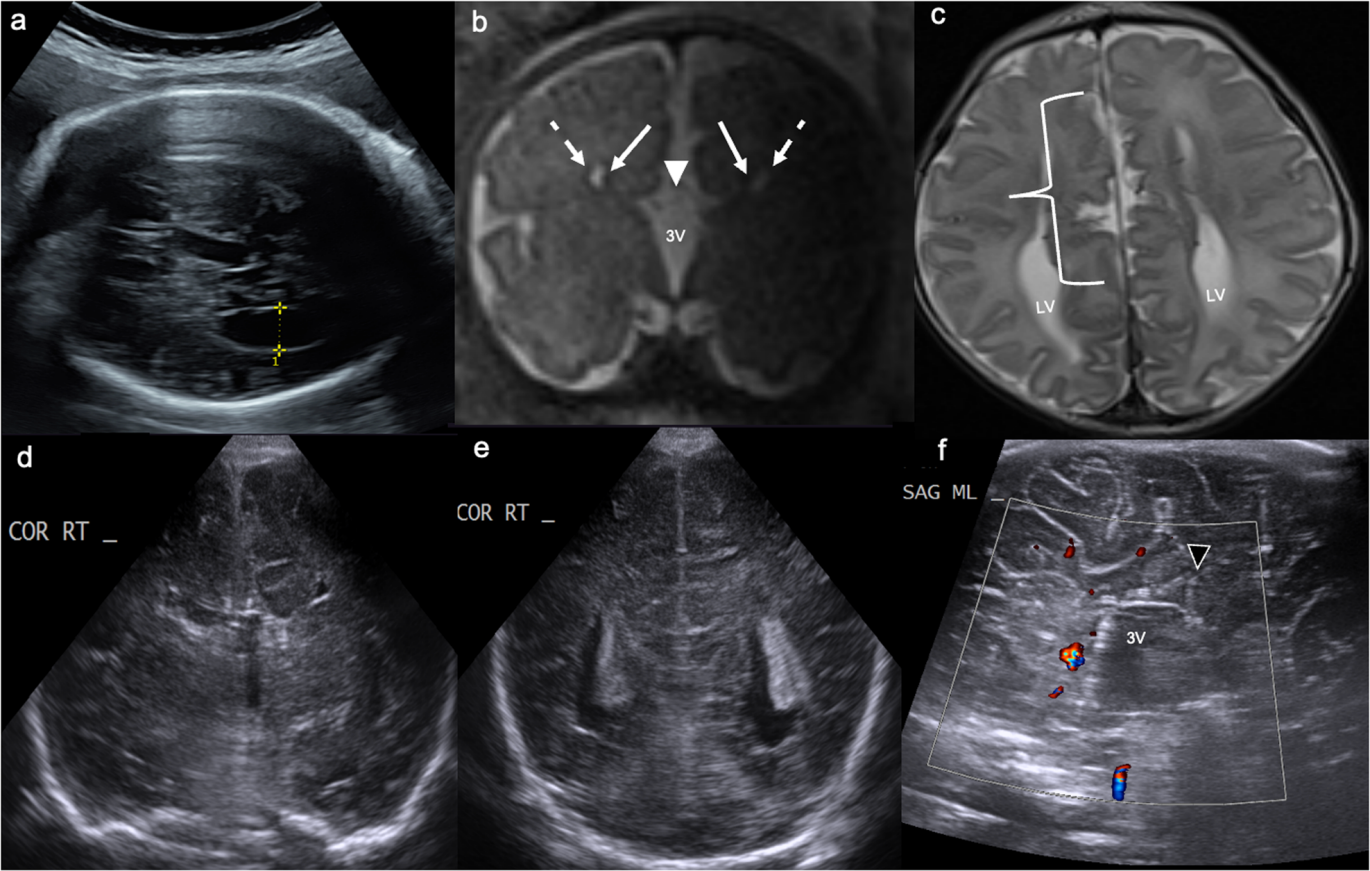
Callosal agenesis. **a** Antenatal ultrasound demonstrates the absence of the septum pellucidum and enlargement of the right lateral ventricular occipital horn (labelled 1). **b** Coronal T2W fetal MRI confirms the absent septum pellucidum, with medially indented (solid arrows) and laterally convex (dashed arrows) frontal horns, and a high-riding third ventricle (3V) extending to the interhemispheric fissure (white arrowhead). **c** Axial T2WI MRI neonatally shows symmetrical enlargement of the lateral ventricular (LV) occipital horns, e.g., colpocephaly, which have a parallel “racing car” configuration; right frontal polymicrogyria is also noted (bracket). **d** Coronal ultrasound at term echoes the fetal MRI findings outlined in **b** above. **e** It also confirms colpocephaly and parallel orientation of the lateral ventricles. **f** Midline sagittal ultrasound demonstrates radially oriented cingulate gyri (black arrowhead) extending to the 3rd ventricle (3V), and no detectable corpus callosum or pericallosal artery

Indirect:

- absence of the septum pellucidum [61].
- lateral convexity of the frontal horns, which are indented medially by Probst bundles (reorganised white matter tracts), resulting in the characteristic “viking helmet”, “steer horn” or “moose head” appearance in the coronal plane.
- parallel, widely-spaced lateral ventricles with dilated atria/occipital horns (“colpocephaly”).
- high-riding third ventricle, which extends to the interhemispheric fissure [60, 61].
- radially oriented cingulate gyri, which extend to the third ventricle.
- abnormal, vertically and posteriorly orientated anterior cerebral arteries (Fig. 9) [61].

Thinning, if suspected on CUS, can be objectively assessed by comparing corpus callosum length and thickness with previously published reference values [63]. Other common associated findings include interhemispheric cysts, lipomas and malformations of cortical development [64], which are better characterised on MRI.

### Tuberous sclerosis

Tuberous sclerosis (TS) is an autosomal dominant neurocutaneous disorder caused by mutations in the TSC1 and TSC2 genes [65]. It results in multiple hamartomas in the brain, skin, kidneys, lungs and heart, which can result in organ dysfunction. Central nervous system (CNS) manifestations are the primary cause of morbidity and mortality [66]. Identification of a cardiac rhabdomyoma on obstetric US is often the first imaging clue to a diagnosis of TS [67]. Characteristic CNS findings of cortical/subcortical tubers and subependymal nodules contribute to a diagnosis of TS in up to 38% of infants. Extensive cortical and subcortical tubers on neonatal CUS have rarely been described [68]. However, sometimes tubers become evident only as myelination progresses [69].

**Fig. 10:**
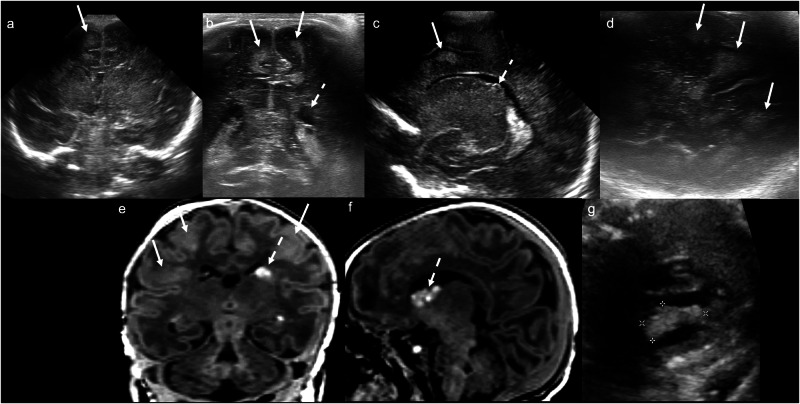
Tuberous sclerosis. Day 1 ultrasound in a 32-week gestation neonate with a paternal history of tuberous sclerosis and an antenatally detected cardiac rhabdomyoma. **a**, **b** Coronal and (**c**, **d**) parasagittal images from day 1 (postnatal) ultrasound demonstrate multiple confluent areas of increased echogenicity in the cortical/subcortical and deep white matter of both cerebral hemispheres (solid arrows), without posterior acoustic shadowing; subependymal nodules are also evident (dashed arrows). **e** Coronal and (**f**) parasagittal T1WI shows the corticosubcortical tubers as subtle, ill-defined T1 hyperintense, radially oriented lesions (solid arrows); the T1 hyperintense subependymal nodules (dashed arrows) are more conspicuous than on CUS. **g** Antenatal ultrasound demonstrated an echogenic intracardiac lesion (measured), consistent with a rhabdomyoma

Cortical and subcortical tubers, which histologically are similar to focal cortical dysplasia type IIb, are visualised on US as thickened, echogenic cortex or subcortical white matter extending along radial migration lines toward the lateral ventricles, perpendicular to the ependyma [70]. Subependymal nodules, which histologically reflect hamartomas, are visualised as iso- to hyperechoic nodules along the lateral ventricular walls, which may be calcified [70]. Subependymal giant cell astrocytomas (SEGA) are World Health Organization (WHO) grade 1 tumours most commonly located at the foramina of Monro and can result in obstructive hydrocephalus. They are also visualised on US as iso- to hyperechoic masses (Fig. 10). It is growth over time and enhancement on post-contrast CT/MRI that differentiates them from subependymal nodules [70]. Extracranial radiological manifestations of TS include lymphangioleiomyomatosis (thoracic and retroperitoneal), renal angiomyolipomas, cysts, and renal cell carcinomas [66].

### (Developmental) megalencephaly

Developmental megalencephaly is a brain overgrowth disorder characterised by an increase in the number and/or size of neurons and glia [71]. It results from PI3K-AKT-MTOR pathway mutations and is seen as part of a spectrum of neurodevelopmental disorders ranging from focal cortical dysplasia to hemimegalencephaly (unilateral cerebral hemispheric overgrowth), megalencephaly (diffuse, bilateral cerebral hemispheric overgrowth) and dysplastic megalencephaly (megalencephaly with cortical malformation) [72]. It can occur in isolation or with other overgrowth syndromes, such as *Proteus* [71].

**Fig. 11:**
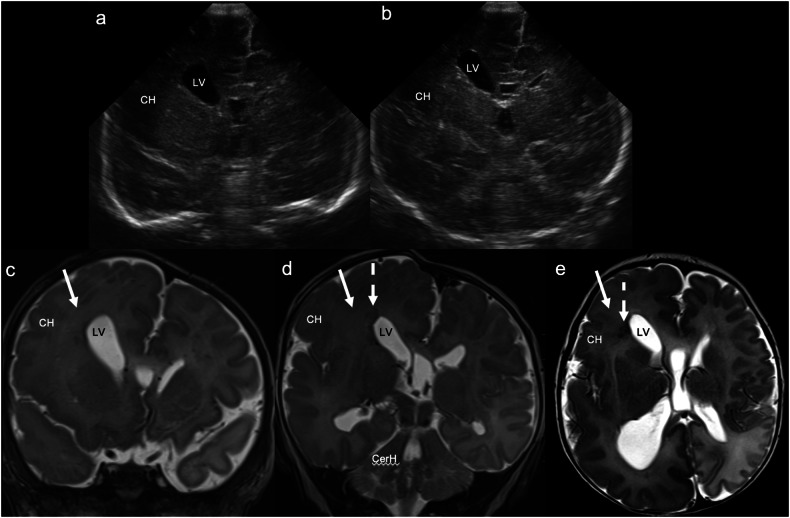
Dysplastic right hemimegalencephaly. Term neonate with antenatal ventriculomegaly, macrocephaly, and an abdominal wall haemangioma evident postnatally. **a**, **b** Coronal images demonstrate unilateral enlargement of the right cerebral hemisphere (CH) and right lateral ventricle (LV), with reduced grey-white matter differentiation in the affected (right) hemisphere. **c**, **d** Coronal and (**e**) axial T2WI confirm unilateral enlargement of the right cerebral hemisphere (CH) (and to a lesser extent the right cerebellar hemisphere (CerH)) and of the right lateral ventricle (LV), with asymmetric T2 low signal in the (dysplastic) white matter of the right cerebral hemisphere (solid arrows) and periventricular nodular heterotopia adjacent to the right frontal horn (dashed arrows). There is also asymmetrical enlargement of the right skull

US findings of “classic” hemimegalencephaly include asymmetric cerebral and sometimes cerebellar hemispheric enlargement, asymmetric enlargement of the ipsilateral lateral ventricle, and asymmetric enlargement of the ipsilateral skull. A straight, superiorly orientated frontal horn may also be evident (Fig. 11) [71, 73, 74]. There may be straightening/flattening or widening of the ipsilateral Sylvian fissure due to opercular underdevelopment [74], although this may be difficult to appreciate on ultrasound. When “dysplastic”, blurring of the grey-white matter junction can also be seen [75]. MRI better characterises cortical malformations and white matter dysplasia [76].

### Lissencephaly-pachygyria

The lissencephaly-pachygyria spectrum is characterised by absent or minimal sulcation and a paucity or lack of normal cortical convolutions, resulting in a smooth brain surface. Lissencephaly is due to abnormal neuronal migration and most commonly occurs due to abnormalities in microtubule structural (i.e., tubulin) or microtubule-associated proteins [77]. The lissencephaly spectrum encompasses agyria (absent gyri), pachygyria (reduced gyri) and subcortical band heterotopia [77]. Pachygyria is also referred to as oligogyria and is typified by a few, broad/thick, simplified gyri with shallow sulci [76]. Subcortical band heterotopia refers to an abnormal (heterotopic), often circumferential, band of subcortical grey matter which is separated from the overlying cerebral cortex and the underlying ventricle by normal white matter [76].

The hallmark of lissencephaly on imaging is cortical thickening and abnormal gyration (Supplementary Fig. 2a–d). Lissencephaly is typically bilateral and symmetrical and usually involves a large portion of the brain. It may be more pronounced anteriorly or posteriorly, which can indicate a specific genetic abnormality—DCX anteriorly, LIS1 posteriorly [76, 78]. It may be associated with other brain malformations, e.g., cerebellar hypoplasia (specifically of the anterior vermis), agenesis of the corpus callosum, basal ganglia dysgenesis, tectal enlargement and brainstem hypoplasia [76]—these are best assessed on MRI.

### Grey matter heterotopia

Grey matter heterotopia (GMH) also occurs due to abnormal neuronal migration. It is characterised by collections of normal neurones in abnormal locations, most commonly in a periventricular/subependymal distribution.

Periventricular (subependymal) GMH is characterised on CUS by hyperechoic periventricular nodules protruding into the ventricles, with irregularity of the ventricular wall [79]. Unlike the subependymal nodules of TS, GMH nodules demonstrate the same echogenicity as grey matter on US and do not calcify (Supplementary Fig. 2e–h). Less commonly, GMH can be subcortical, affecting the white matter of the cerebral hemisphere. The term “subcortical” is misleading as this form of GMH can occur in either the subcortical or deep white matter, or can extend from the ventricular surface to the overlying (often dysplastic) cortex in a transmantle distribution [76]. This is visualised on CUS as focal, abnormal grey matter echogenicity in the affected white matter [79]. Finally, subcortical band heterotopia, a subtype of lissencephaly, describes a circumferential, band-like form of GMH deep to the cortical mantle, which is often bilateral and symmetrical [76]. This may be difficult to detect sonographically.

## Tumours

Congenital brain tumours, defined as those diagnosed before 6 months of age, are rare and account for < 2% of paediatric brain tumours [80]. They are most commonly supratentorial and include gliomas (high- and low-grade), medulloblastomas, choroid plexus, atypical teratoid/rhabdoid, germ cell and glioneuronal tumours [80].

### Choroid plexus tumours

Choroid plexus neoplasms are rare tumours that are comparatively more common in neonates [81]. While the majority are sporadic, they can be associated with Li Fraumeni [82] and Aicardi syndromes [83]. Choroid plexus neoplasms consist mainly of WHO grade 1 papillomas (approximately 80%). Less commonly, WHO grade 2 atypical papillomas or grade 3 carcinomas occur [81]. Papillomas arise from the choroid plexus epithelium, are typically intraventricular in location and are most often found in the lateral ventricular trigones [84].

**Fig. 12:**
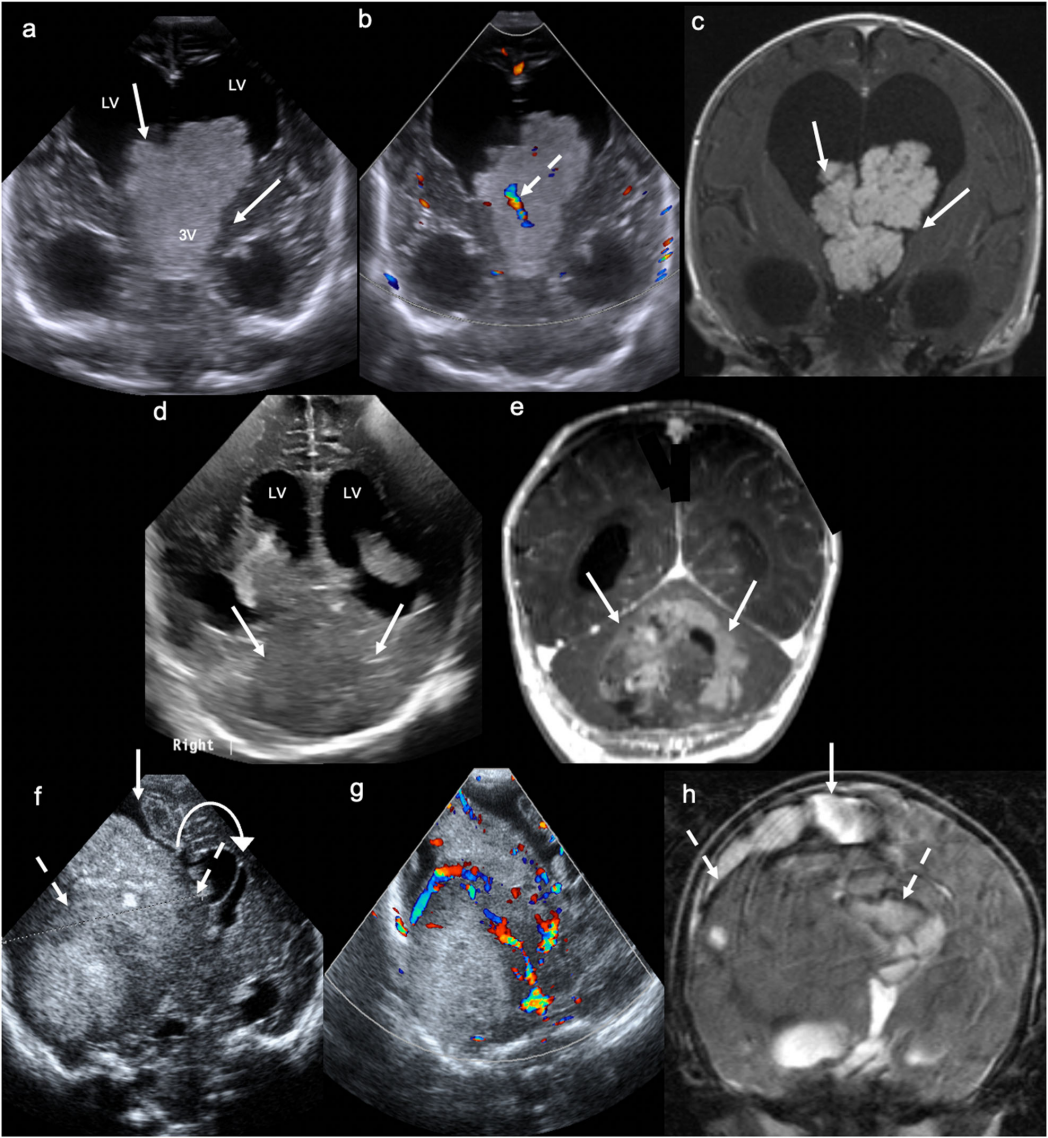
Choroid plexus papilloma. Ultrasound at 2 months in an infant with rapidly increasing head circumference and sun-setting eyes. **a** Coronal image demonstrates a large, lobulated/cauliflower-like, echogenic, non-shadowing mass (solid arrows) occupying the lateral (LV) and third ventricles (3V), with associated hydrocephalus. **b** Coronal colour Doppler image demonstrates a vascular pedicle centrally within the mass (dashed arrow), which had an arterial waveform on spectral Doppler. **c** Coronal post-contrast T1WI shows avid enhancement within the large solid intraventricular mass (solid arrows), with associated hydrocephalus. This lesion proved to be a choroid plexus papilloma. Atypical teratoid/rhabdoid tumour. Ultrasound at 4 months in an infant with reduced feeding, vomiting, and increasing head circumference. The patient had a normal head ultrasound on day 1 of life. **d** Coronal image demonstrates a large, ill-defined, and heterogeneous posterior fossa mass (solid arrows), with obstructive hydrocephalus involving the lateral ventricles (LV). **e** Coronal post-contrast T1WI confirms the large, solid, heterogeneously enhancing and obstructing posterior fossa mass (solid arrows). This lesion proved to be an atypical teratoid/rhabdoid tumour. Desmoplastic infantile ganglioglioma. Ultrasound at 2 weeks in a neonate presenting with floppiness and increasing head circumference. The patient had a normal MRI brain on day 1 of life. **f** Coronal image demonstrates a large, heterogeneous mass occupying most of the right cerebral hemisphere, with anechoic cystic (solid arrow) and echogenic solid (dashed arrows) components. There is an associated leftward midline shift (curved arrow). **g** Coronal oblique colour Doppler image demonstrates marked vascularity within the mass. **h** Coronal T2WI confirms the large mixed cystic (solid arrow) and solid (dashed arrows) mass, with obstructive hydrocephalus. This lesion proved to be a desmoplastic infantile ganglioglioma

CUS demonstrates an echogenic, lobulated/cauliflower-like intraventricular mass that is highly vascular, often with large choroidal arterial feeders and large draining veins [15]. There is typically associated hydrocephalus due to CSF overproduction and mechanical obstruction of CSF flow by the mass [85]. (Fig. 12a–c). Choroid plexus tumours, being echogenic intraventricular lesions, can be misdiagnosed as intraventricular haemorrhage (IVH). However, the abundant vascularity that characterises choroid plexus tumours, as well as their lack of evolution over time, helps to differentiate them from IVH. The prognosis of choroid plexus papillomas is excellent, with post-surgical resection 6-year survival rates of up to 100% [86]. However, choroid plexus tumours can seed via CSF [87], which is unsurprisingly associated with a poorer prognosis [86]. Pre-operative embolisation may decrease blood loss, morbidity and mortality, and increase the likelihood of gross total resection [88].

### Atypical teratoid/rhabdoid tumours

Atypical teratoid/rhabdoid tumours (ATRTs), like medulloblastomas, are aggressive WHO grade 4 embryonal tumours characterised histologically by dense small round blue cells and poorly differentiated rhabdoid cells [89]. They tend to occur at a younger age than medulloblastomas (usually before 3 years and within the first year of life in one third) and portend a poorer prognosis [89]. They are associated with mutations in the SMARCB1/IN1 gene [90] and can be seen in conjunction with rhabdoid tumours elsewhere in the CNS, kidneys, orbits, neck, liver and extremities [91]. Over half of CNS ATRTs occur in the paramedian infratentorial compartment or cerebellopontine angle [89, 92].

While there is little in the literature regarding the CUS findings of ATRT, lesions are typically large and heterogeneous with haemorrhage, calcifications, cysts and/or necrosis (Fig. 12d, e) [93]. Treatment consists of surgical resection, with or without adjuvant radiotherapy, high-dose chemotherapy and autologous stem cell transplantation. ATRTs are aggressive tumours with a dismal prognosis [94].

### Desmoplastic infantile ganglioglioma

Desmoplastic infantile gangliogliomas are very rare tumours that typically occur in infants, but sometimes congenitally [95]. They are WHO grade I tumours [96]. They tend to be large, predominantly cystic, often with a solid peripheral nodule, and occur almost exclusively in a supratentorial/cerebral hemispheric location [97].

They are visualised on US as large, multicystic/hypoechoic masses with a hyperechoic solid component [98] (Fig. 12f–h). Treatment is with surgical resection, which may be complicated by young patient age, low patient weight, large tumour size and high tumour vascularity [95]. Long-term prognosis is favourable providing resection is complete [95].

## Trauma

### Birth-related trauma

Mechanical birth-related trauma describes injuries sustained during the birthing process. Most birth-related trauma is to the head—scalp injuries comprise most cases; however, extra- and intra-axial haemorrhage may also occur [99]. Most injuries are mild and transient, but severe cases can lead to disability and even death. There are three main types of extracranial/scalp haematomas: caput succedaneum, which occur deep to the subcutaneous connective tissues of the scalp and superficial to the galea aponeurosis, these are not bound by sutures and occur most commonly at the vertex; subgaleal haematomas, which occur deep to the galea but superficial to the periosteum of the skull, and are not confined by sutures; and cephalohaematomas, which occur deep to the periosteum of the skull, and are thus bound by sutures [100, 101]. These are usually diagnosed clinically and of little consequence, except in the case of large subgaleal haematomas, where voluminous blood loss into the subgaleal space can result in neonatal death and/or where cosmetic deformity ensues [102].

All of these haematomas are visualised on CUS as hypoechoic fluid collections. Cephalohaematomas are bound by sutures, whereas subgaleal haematomas can spread and cross suture boundaries, helping to differentiate between these two entities (Supplementary Fig. 3). If persistent, cephalohaematomas and subgaleal haematomas can peripherally calcify, appearing as complex fluid collections with punctate linear echogenic foci superficially on CUS [103]. They resolve by gradual calcification, with eventual incorporation into the underlying skull.

Skull fractures are rare in the setting of mechanical birth trauma. If they occur, they are typically linear, depressed or diastatic [103], and may be associated with extra- and intracranial haemorrhage. While skull fractures are best imaged with CT, they may be visualised on high-frequency CUS [104, 105] as a focal discontinuity in the cortex, with adjacent soft tissue swelling.

Birth-related intracranial haemorrhage (ICH) is common and may be incidentally detected on MRI in up to 59% of term neonates. It is especially common post-vaginal delivery [106]. It can occur extra-axially within the epidural, subdural and/or subarachnoid spaces, or intra-axially within the cerebral or cerebellar parenchyma [103]. When asymptomatic, it is most commonly subdural and/or subarachnoid [106].

Cross-sectional imaging, notably MRI with susceptibility-weighted imaging, is far superior to CUS in the detection of small volume subdural, infratentorial haemorrhage. However, US, due to its accessibility and portability, may be the first to diagnose or suspect ICH in the context of birth-related trauma. Extra-axial haemorrhage is visualised on CUS as a peripheral hypoechoic fluid collection [103]. Acute intra-axial haemorrhage is visualised as an echogenic parenchymal lesion, occasionally with associated mass effect [107].

### Inflicted injury

Physical child abuse is insidious, transcends racial and socio-economic groups, can present at any time, and in an unrelated context [108, 109]. CT and MRI are the primary neuroimaging modalities employed in children with suspected abusive head trauma (AHT)/inflicted injury (II) [110]. However, SDH, which is the most common intracranial manifestation of AHT [111], may be seen in infants in whom CUS has been performed for other clinical indications [112].

The main CUS finding of II/AHT is subdural collections, with inward displacement of the linear, echogenic arachnoid membrane (which is not visible as a discrete structure in the absence of a subdural collection) and of subarachnoid vessels toward the cortical surface. Subdural collections may be uni- or multifocal, homogeneous or heterogeneous, and uni- or multicompartmental/septated (Supplementary Fig. 4) [113]. There may be mass effect on the underlying brain [113, 114], eventually resulting in brain atrophy [112]. Imaging with a high-frequency linear array transducer and colour Doppler is crucial. If subdural effusions are identified, cross-sectional imaging is typically performed [110] and may reveal, in addition to SDH, rupture/thrombosis of bridging veins, skull fractures/scalp swelling, and parenchymal injury, i.e., cerebral contusions, lacerations and/or hypoxic ischaemic injury [110]. If brain imaging findings are suspicious for II, an MRI of the spine should be performed to screen the remainder of the neuraxis. It is worth noting that, as in the brain, spinal SDH may be visualised on US performed for an unrelated indication, e.g., suspected spinal dysraphism. Spinal SDH is strongly associated with AHT [110] and necessitates an urgent paediatric referral.

## Future directions

With ever-improving equipment, the spectrum of pathologies that can be diagnosed with CUS continues to expand. High-frequency linear transducers allow increased visualisation of near-field and midline structures, thereby increasing diagnostic capabilities [115]. Specialised US techniques, like contrast-enhanced ultrasound (CEUS), ultrafast Doppler and elastography may also add value to conventional US.

CEUS involves the intravenous injection of microbubbles to assess perfusion. US contrast is approved by the United States Food and Drug Administration for the characterisation of liver lesions in children. Although not yet approved for use in the brain, it has been shown to be safe in a small neonatal cohort [116]. CEUS assesses for areas of symmetric or focally abnormal perfusion in the brain. Potential scenarios in which it may be helpful include: diagnosis and monitoring of HIE, arterial stroke (both hypoperfusion and luxury hyperperfusion) and brain death; assessing residual blood flow in vein of Galen aneurysmal malformations post-embolisation; assessing dural venous sinus patency; differentiating haemorrhage from tumour; and live perfusion monitoring in the setting of cardiac surgery [116].

Ultrafast Doppler, with its high frame rate and increased sensitivity for blood flow, has facilitated visualisation of previously imperceptible blood vessels, thereby allowing quantification of perfusion and resistivity mapping in the neonatal brain [117]. It also permits functional imaging, whereby small changes in blood volume act as a surrogate for brain activity [118]. Given its high spatial and temporal resolution, deep brain penetration, non-invasiveness, and portability, it holds promise as a valuable tool for the future detection of abnormal brain activity, particularly in the setting of HIE [117].

Although not yet approved for use in the neonatal brain, shear wave elastography, which measures tissue stiffness, may also play a role in detecting brain injury in the future [119]. However, the safety and potential bioeffects of these new sonographic techniques on the developing brain must always be considered prior to clinical implementation.

Perinatal post mortem ultrasound (PMUS) is a technique that, until recently, has been poorly described in the literature. However, with rates of consent for conventional autopsy declining and limited availability for post mortem CT and MRI, it may prove a reasonable alternative [120]. Congenital CNS abnormalities are the most common structural reason for termination of pregnancy. The brain and spine are well-imaged on PMUS, allowing detection of neural tube defects, callosal and neuronal migration anomalies, and congenital tumours, amongst others [120]. Thus, perinatal PMUS could play a role in CNS “screening”, especially when post mortem MRI is unavailable.

## Conclusion

Through this comprehensive case review, we illustrate various CNS pathologies affecting term neonates and infants, including vascular lesions, infection, genetic disorders/malformations, tumours and trauma: some of these pathologies have rarely, if ever, been described on CUS. US is an excellent initial imaging modality in the assessment of the developing term neonatal and infantile brain. Given its widespread accessibility and availability, low cost, speed, portability, safety profile, and ease of repetition, it has certain advantages over MRI, which remains the gold standard for the diagnosis of many of the pathologies illustrated herein and remains complementary. Amidst ever-advancing technology, CUS continues to serve as an invaluable diagnostic tool, while holding promise in future clinical applications.

## Supplementary information

ELECTRONIC SUPPLEMENTARY MATERIAL

## Data Availability

The data will not be made publicly available due to privacy restrictions.

## Abbreviations

AHT: Abusive head trauma
AIS: Arterial ischaemic stroke
ATRT: Atypical teratoid rhabdoid tumour
CEUS: Contrast-enhanced ultrasound
CMV: Cytomegalovirus
CNS: Central nervous system
CSF: Cerebrospinal fluid
CSVT: Cerebral sinovenous thrombosis
CUS: Cranial US
DSM: Dural sinus malformation
GMH: Grey matter heterotopia
HIE: Hypoxic ischaemic encephalopathy
ICH: Intracranial haemorrhage
II: Inflicted injury
IVH: Intraventricular haemorrhage
PMUS: Post mortem ultrasound.
RI: Resistive index
SAH: Subarachnoid haemorrhage
SDH: Subdural haemorrhage/haematoma
TS: Tuberous sclerosis
VoGM: Vein of Galen aneurysmal malformation
WHO: World Health Organization

## Declarations

### Guarantor

Dr Rahul Lakshmanan, Medical Imaging Department, Perth Children’s Hospital, 15 Hospital Avenue, Nedlands, WA 6009, Australia. Telephone: +61 8 6456 2222. Facsimile: +61 6 6456 0072. E-mail: rahul.lakshmanan@health.wa.gov.au.
