# Supplementary material for: Paediatric cranial ultrasound: abnormalities of the brain in term neonates and young infants
Source: Insights Imaging. 2025 Jul 22;16:159. doi: 10.1186/s13244-025-02031-4 (PMC12283534; doi:10.1186/s13244-025-02031-4)
Supplement: Supplementary file 1 — ELECTRONIC SUPPLEMENTARY MATERIAL [file 13244_2025_2031_MOESM1_ESM.pdf]

# Paediatric cranial ultrasound: Abnormalities of the brain in term neonates and young infants

## ELECTRONIC SUPPLEMENTARY MATERIAL

Insights Imaging (2025) Leidhin CN, Paddock M, Parizel PM, Warne RR, Shipman P, Lakshmanan R.

**Supplementary figure 1.** Dural sinus malformation.

*Day 1 ultrasound in a term neonate post-vacuum assisted delivery, screening for intraventricular haemorrhage.*

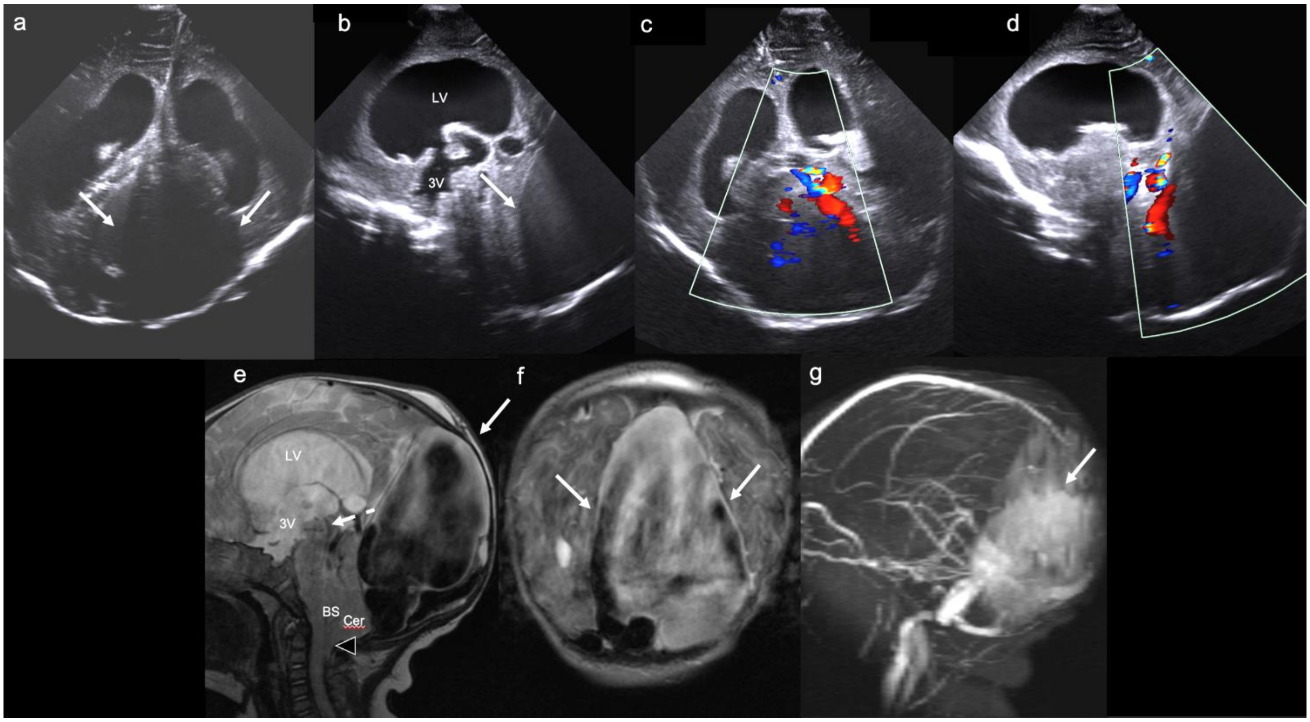


(a) Coronal image demonstrates a hypoechoic mass with low-level internal echoes, which appears centered on the tentorium cerebelli and involving the torcular herophili/confluence of sinuses (solid arrows).

(b) Midline sagittal image confirms that the hypoechoic mass (solid arrow) is centered on the torcular herophili. The lateral ventricle (LV) is dilated.

(c) Coronal and (d) midline sagittal colour Doppler images demonstrate flow in the anterior wall of the mass.

(e) Midline sagittal and (f) posterior coronal T2WI demonstrate gross, sac-like enlargement of the torcula (solid arrows), which has a triangular morphology coronally (f). There is mixed T2 high and low signal within the mass, indicating blood products of differing ages.

Note the enlargement of the lateral (LV) and third ventricles (3V) due to obstruction of the aqueduct of Sylvius (dashed arrow) by the mass.

Note compression of the cerebellum (Cer) and brainstem (BS), with cerebellar tonsillar descent (black arrowhead) due to mass effect. The fourth ventricle is effaced.

(g) Midline sagittal MRV demonstrates slow (venous) flow within the mass (solid arrow), which abuts the superior sagittal sinus superiorly and the medial transverse sinuses inferiorly.

**Supplementary figure 2.** Lissencephaly-pachygyria (a-d).

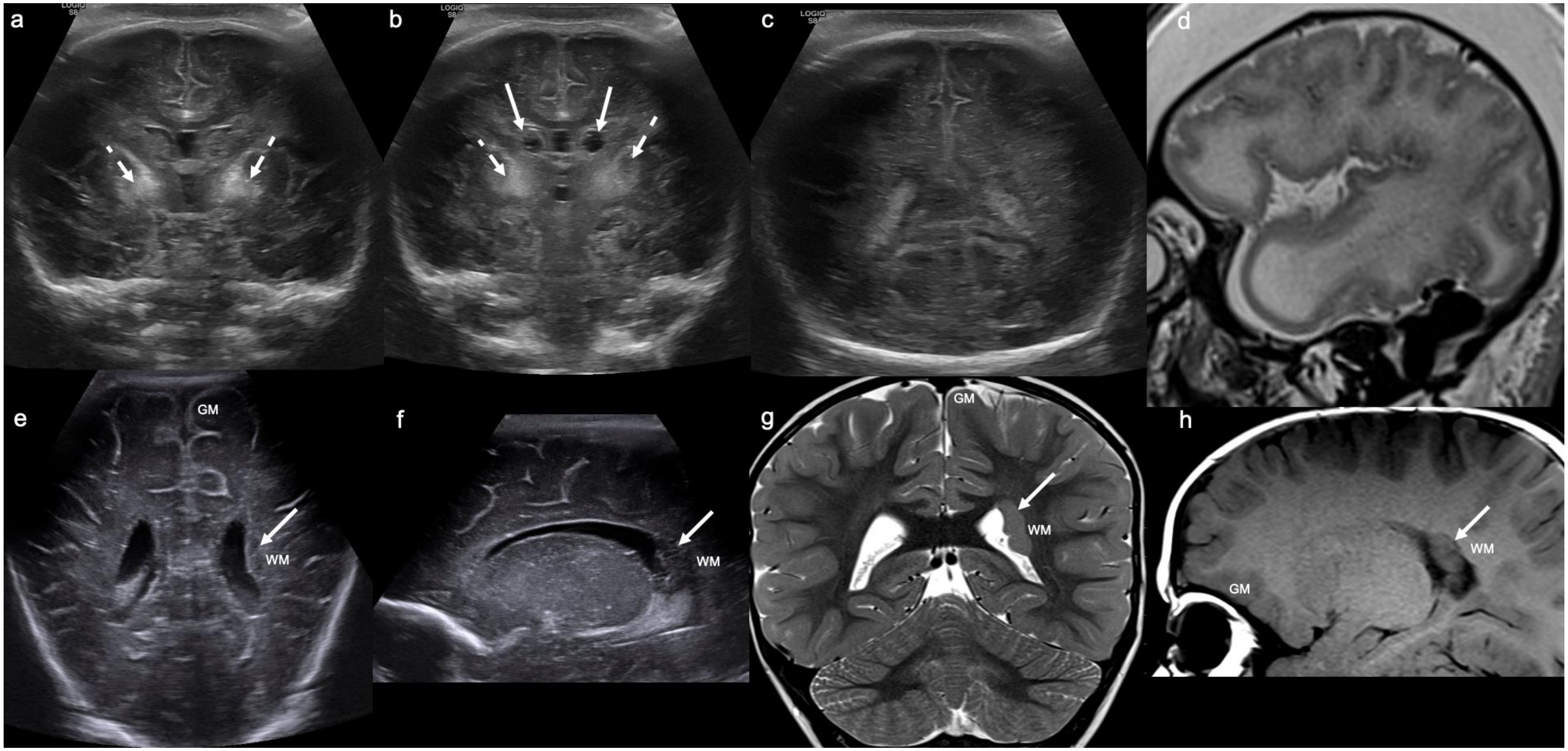


*Term neonate with seizures and dysmorphic features.* (a-c) Coronal images demonstrate a diffusely abnormal, smooth/poorly-sulcated brain. *The term brain should have sulci deeper than the gyri are wide, with secondary sulcation (i.e. insular, cingulate and occipital sulci) also present.* Also note bilateral germinolytic cysts (solid arrows) and bilateral, symmetrical increased echogenicity of the globi pallidi (dashed arrows).

(d) Sagittal T2WI (performed post-mortem) confirms lissencephaly-pachygyria with an anterior to posterior gradient.

Periventricular nodular grey matter heterotopia (e-h).

*Ultrasound at 6 weeks in an infant with seizures.*

(e) Coronal and (f) left parasagittal images demonstrate a nodule indenting the posterior body of the left lateral ventricle (solid arrows), which is hypoechoic relative to the adjacent periventricular white matter (WM) and isoechoic to grey matter (GM).

(g) Coronal T2WI and (h) left parasagittal T1WI confirm confluent periventricular nodules (solid arrows), which are isointense to GM.

**Supplementary figure 3.** Birth-related trauma.

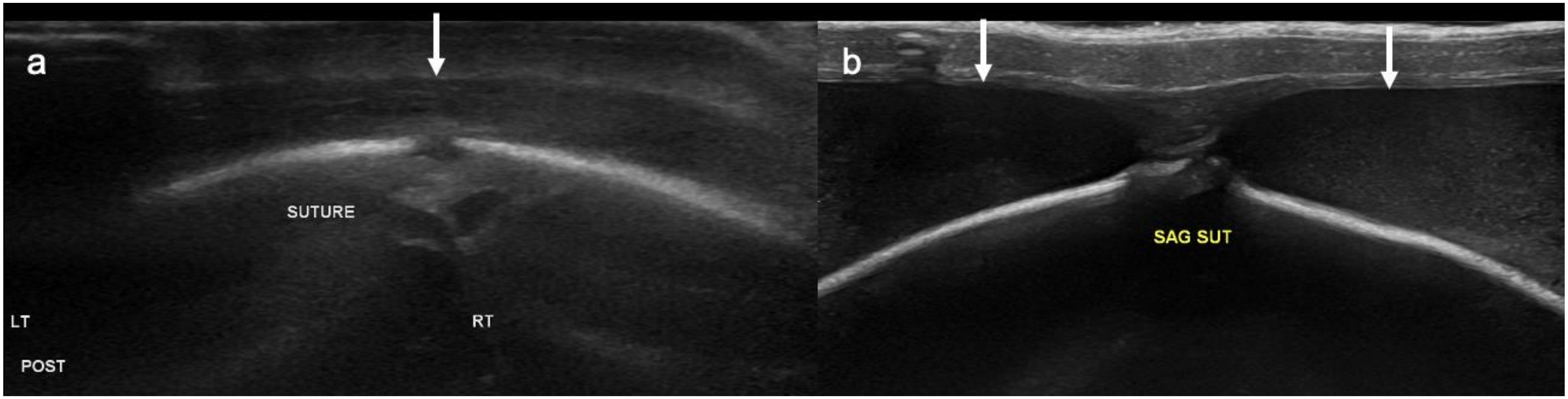


*Ultrasound at day 1 of life in a term neonate born via vacuum-assisted delivery.*

(a) High-frequency linear probe demonstrates a large, hypoechoic posterior scalp collection, which crosses the right lambdoid suture, consistent with a subgaleal haematoma.

*Ultrasound at day 15 of life in a term neonate born via vacuum-assisted delivery.*

(b) High-frequency linear probe demonstrates bilateral, anechoic paramidline collections, which are bound by the sagittal suture, consistent with bilateral cephalohaematomas.

**Supplementary figure 4.** Inflicted injury.

*Ultrasound at 2 months in an infant with suspected cluster seizures and decreased hemoglobin level.*

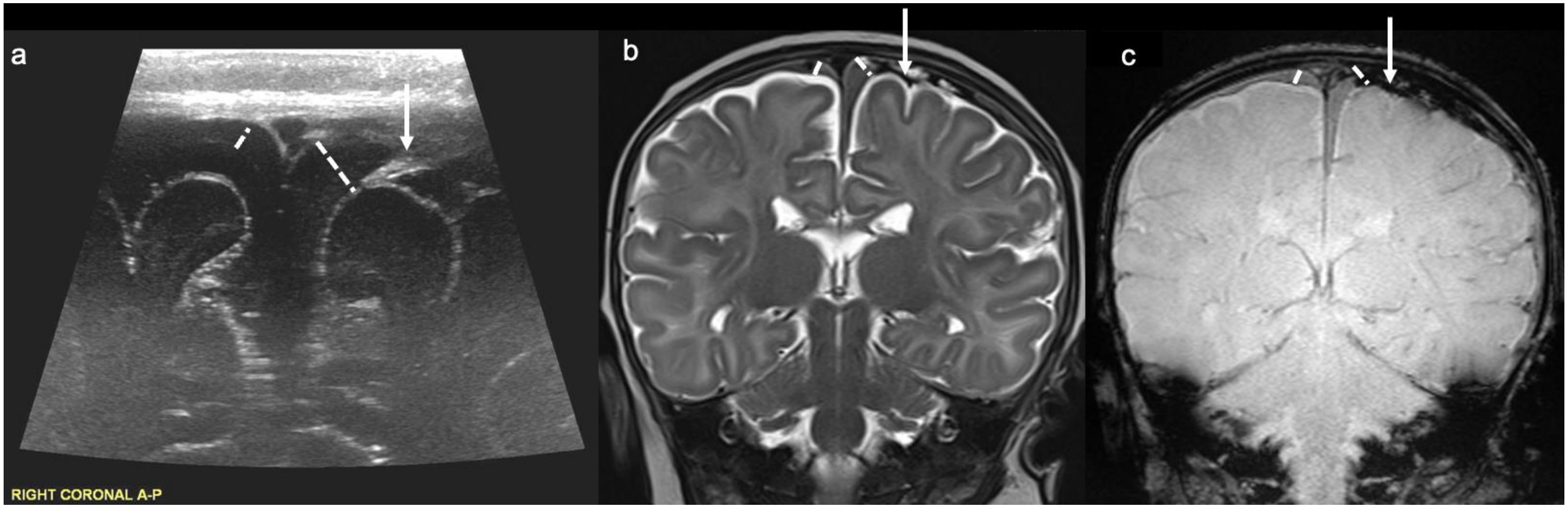


(a) Coronal image with a high-frequency linear probe demonstrates bilateral hypoechoic parafalcine subdural effusions (dashed lines), containing echogenic material. A linear echogenic structure (solid arrow) is seen traversing the left extra-axial space.

(b) Coronal T2WI and (c) gradient echo MRI confirm bilateral parafalcine subdural haemorrhages (dashed lines). The linear echogenic structure within the left extra-axial space on US is of low signal with susceptibility artefact on MRI; it bridges the subarachnoid space and communicates with the left parafalcine subdural collection (solid arrows). This is consistent with a thrombosed cortical vein.
